# Identification and Subtyping of *Salmonella* Isolates Using Matrix-Assisted Laser Desorption–Ionization Time-of-Flight Mass Spectrometry (MALDI-TOF)

**DOI:** 10.3390/microorganisms10040688

**Published:** 2022-03-23

**Authors:** Anil K. Persad, Hanan A. Fahmy, Nicholas Anderson, Jing Cui, Zeynal Topalcengiz, Saharuetai Jeamsripong, Patrick M. Spanninger, Robert L. Buchanan, Kalmia E. Kniel, Michele T. Jay-Russell, Michelle D. Danyluk, Gireesh Rajashekara, Jeffrey T. LeJeune

**Affiliations:** 1 Center for Food Animal Health, Department of Animal Sciences, Ohio Agricultural Research and Development Center, The Ohio State University, Wooster, OH 44691, USA; anilkpersad@gmail.com (A.K.P.); dr.hanana.fahmy@hotmail.com (H.A.F.); anderson.3052@osu.edu (N.A.); rajashekara.2@osu.edu (G.R.); 2 School of Veterinary Medicine, Faculty of Medical Sciences, The University of the West Indies, Eric Williams Medical Sciences Complex, Mt. Hope, Trinidad and Tobago, West Indies; 3 Animal Health Research Institute (AHRI), Giza 12618, Egypt; 4 Animal Disease Diagnostic Laboratory, Ohio Department of Agriculture, 8995 East Main St., Building 6, Reynoldsburg, OH 43068, USA; jing.cui@agri.ohio.gov; 5 Department of Food Engineering, Faculty of Engineering and Architecture, Muş Alparslan University, Muş 49250, Turkey; zeynaltopalcengiz@gmail.com; 6 Department of Food Science and Human Nutrition, Citrus Research and Education Center, University of Florida, Lake Alfred, FL 33850, USA; mddanyluk@ufl.edu; 7 Western Institute for Food Safety and Security, UC Davis, Davis, CA 95618, USA; sjeamsripong@ucdavis.edu (S.J.); mjay@ucdavis.edu (M.T.J.-R.); 8 Research Unit in Microbial Food Safety and Antimicrobial Resistance, Department of Veterinary Public Health, Faculty of Veterinary Science, Chulalongkorn University, Pathumwan, Bangkok 10330, Thailand; 9 Department of Animal and Food Sciences, University of Delaware, Newark, DE 19716, USA; pspannin@udel.edu (P.M.S.); kniel@udel.edu (K.E.K.); 10 Department of Nutrition and Food Science and Center for Food Safety and Security Systems, University of Maryland, College Park, MA 20742, USA; rbuchana@umd.edu

**Keywords:** mass spectrometry, matrix-assisted laser desorption–ionization, *Salmonella*, subtyping, serology, MALDI-TOF

## Abstract

Subtyping of bacterial isolates of the same genus and species is an important tool in epidemiological investigations. A number of phenotypic and genotypic subtyping methods are available; however, most of these methods are labor-intensive and time-consuming and require considerable operator skill and a wealth of reagents. Matrix-Assisted Laser Desorption–Ionization Time-of-Flight Mass Spectrometry (MALDI-TOF), an alternative to conventional subtyping methods, offers a rapid, reproducible method for bacterial identification with a high sensitivity and specificity and at minimal cost. The purpose of this study was to determine the feasibility of using MALDI-TOF to differentiate between six *Salmonella* serovars recovered from experimental microcosms inoculated with known strains of *Salmonella*. Following the establishment of a MALDI-TOF reference library for this project, the identity of 843 *Salmonella* isolates recovered from these microcosms was assessed using both MALDI-TOF and conventional methods (serotyping/PCR). All 843 isolates were identified as being *Salmonella* species. Overall, 803/843 (95%) of these isolates were identified similarly using the two different methods. Positive percent agreement at the serovar level ranged from 79 to 100%, and negative percent agreement for all serovars was greater than 98%. Cohen’s kappa ranged from 0.85 to 0.98 for the different serovars. This study demonstrates that MALDI-TOF is a viable alternative for the rapid identification and differentiation of *Salmonella* serovars.

## 1. Introduction

*Salmonella enterica* serovars have been associated with foodborne disease globally for over 100 years [1]. While the global incidence is unknown, within the United States the disease burden is estimated to be over one million cases annually, with 16,000 hospitalizations and almost 500 deaths [2]. Most human cases are self-limiting; however, young children under five years, the elderly, and the immunocompromised are at the highest risk of becoming infected and developing complications [3]. Humans can be infected through the consumption of contaminated food and water, direct contact with infected animals and their environment, or via person-to-person transmission. A necessity in the successful treatment, prevention, and control of foodborne disease outbreaks is the rapid and accurate identification of the offending pathogen [4].

Bacterial subtyping of isolates of the same genus and species is an important tool in disease surveillance, outbreak investigations, and epidemiological research. A number of phenotypic and genotypic subtyping methods are available [5]; however, for foodborne pathogens, such as *Salmonella enterica* subsp. *enterica*, serotyping is often among the most widely used initial characterizations performed on isolates. *Salmonella* serotyping is a phenotypic subtyping method that has been in existence for over 80 years and is still the primary screening method in many laboratories [6]. The basis of this method of subtyping is observation for agglutination reactions occurring between specific antisera and somatic (O) and flagellar (H) antigens of the *Salmonella* isolate. The *Salmonella* isolate is then classified using the Kauffman–White–LeMinor scheme [7]. However, since there are over 2500 serovars of *Salmonella enterica*, with 46 O antigens and 114 H variations [8], serotyping can be quite exhausting and time-consuming and require a vast number of antisera [9]. Additionally, the possibility exists for inaccurate classification as a result of observer’s error, nonspecific agglutination, auto-agglutination, or loss of antigenic expression [6,10].

In comparison, Matrix Laser Desorption–Ionization Time of Flight (MALDI–TOF), a library-based approach to bacterial identification, offers a rapid, reproducible method for bacterial identification with a high sensitivity and specificity and at minimal cost [11,12]. MALDI-TOF uses the mass-to-charge ratio profile of bacterial microbial proteins and peptides for bacterial identification [13]. This mass-to-charge ratio profile or mass spectral profile analysis is usually confined to the 2–20 kDA range, since the majority of peaks in this range are representative of ribosomal proteins which are less influenced by variability in cultivation conditions [14]. Bacterial isolates can be characterized at the genus and species level via the identification of a unique biomarker ion peak(s) or by matching the mass spectral profile or “fingerprint” of query bacteria with the spectral profiles of known bacterial species within the established MALDI-TOF library using pattern recognition algorithms [5,9,11,15]. Identification at the subspecies level can be more difficult due to the lack of unique ion peaks between serotypes and also due to poor differentiation of mass spectral profiles between serotypes [13]. Although MALDI-TOF use has been well validated for bacterial identification at the species level, differentiation at the subspecies level is less well described.

The purpose of this study was to determine the feasibility of using MALDI-TOF technology to differentiate six known *Salmonella* serovars recovered from a long-term fecal survival study. To achieve this, we (1) constructed and validates reference spectra for the six Salmonella serovars and (2) compared the results obtained from MALDI-TOF subtyping with those from conventional subtyping of *Salmonella* isolates recovered from experimental microcosms inoculated with these same strains of *Salmonella enterica* serovars.

## 2. Materials and Methods

**Bacterial strains**: The *Salmonella enterica* serovars used in this study were *S.* Anatum (K2669 CDC clinical isolate), *S.* Braenderup (04E61556), *S.* Javiana (ATCC^®^ BAA-1593^TM^), S. Montevideo (Human–tomato linked), *S.* Newport (Environmental isolate), and *S.* Typhimurium (ATCC^®^ 700720^TM^). The serovars and their epidemiological history were kindly provided by Dr. Michelle Danyluk, Citrus Research and Education Center, University of Florida. The six *Salmonella* serovars were transformed to exhibit Rifampicin resistance at 80 µg/mL in order to distinguish the inoculated serovars from background microflora in feces. Rifampicin resistance was achieved by serial passage of the parent salmonella isolates in increasing concentrations (10, 20, 30, 40, 50, 60, 70, 80, 90, 100 µg/mL) of Rifampicin (Fischer Scientific, Fair lawn, NJ, USA). The final broth culture was plated on LB agar with rifampicin (80 µg/mL) and incubated overnight at 37 °C. The next day, three isolates were selected and grown overnight in BHI broth and stored at −80 °C with 30% buffered glycerol for use at a later time. Rifampicin resistance is conferred as a result of mutation in the beta subunit of RNA polymerase. Previous studies comparing the use of plasmids versus chromosomal mutations for bacterial identification in long-term survival studies indicated that bacteria with chromosomal mutations accurately represented the bacterial population, and this method was much more reliable than using plasmids [16].

**Experimental microcosm:** The fecal survival study was conducted at four different laboratories in California, Delaware, Florida, and Ohio as described in Topalcengiz et al. (2020) [15]. Briefly, the rifampicin-resistant serovars were cultured separately overnight at 37 °C in Buffered Peptone Water (BPW, Acumedia, East Lansing, MI, USA). Following incubation, 45 mL samples of each Salmonella broth culture was placed in 50 mL centrifuge tubes and centrifuged at 4600× *g* for 20 min. The supernatant was decanted, and the bacterial pellet was resuspended in 45 mL of 1× phosphate-buffered saline (PBS, AMRESCO, Salon, OH, USA). This ‘washing’ procedure was repeated two more times to ensure the removal of any nutrient content or antibiotic selective pressure. After the third washing and re-suspension of the bacterial pellet, the optical densities of the resuspended solutions were measured to attain an inoculation dose between 10^4^ and 10^5^ CFU/gram feces, and the feces were inoculated. The inoculated fecal microcosms were left at room temperature (22 ± 3 °C) until the day of sampling. The population of *Salmonella* in each fecal sample (cattle, deer, raccoon, wild hog, and waterfowl) was enumerated at days 1, 3, 5, 7, 14 and monthly by surface plating 1 mL dilutions (10^−1^–10^−6^) of each fecal sample on LB agar, Lennox (LB, Acumedia, East Lansing, MI, USA) plates containing 80 µg/mL of rifampicin and 50 µg/mL of cycloheximide (Sigma–Aldrich, St. Louis, MO, USA).Up to 10 (if present) *Salmonella* colonies recovered from each fecal sample on every sampling day were transferred from the LB agar plates to 2.0 mL centrifuge tubes containing 1.0 mL of brain heart infusion (BHI, Acumedia, East Lansing, MI, USA) and cultured overnight at 37 °C. The next day, 300 μL of buffered glycerol (VWR International, Radnor, PA, USA) was added to each tube, and the content mixed. These cultures were then stored at −80 °C until identification. The recovered *Salmonella* isolates from California, Florida, and Delaware were shipped to the Ohio Agriculture Research and Development Center (OARDC) for identification.

**Construction of Reference Spectra**: The reference *Salmonella* strains were streaked onto XLT-4 agar (Acumedia, East Lansing, MI, USA) plates and incubated at 37 °C for 18–24 h. Following incubation, one colony from each plate was selected and streaked onto LB agar plates supplemented with rifampicin (80 µg/mL) and incubated at 37 °C for 18–24 h. Following incubation, 15 colonies were selected from each serotype and subjected to protein extraction procedures as described by the manufacturer [16]. Briefly, each colony was transferred from the agar plate to a 1.7 mL microcentrifuge tube containing 300 µL of HPLC-grade water (EMD Chemicals–Gibbstown, NJ, USA). The contents of the tube were mixed thoroughly by vortexing for approximately 1 min. Following thorough mixing, 900 µL of HPLC grade Ethanol (ACROS Organics, Fischer Scientific, Fair Lawn, NJ, USA) was added to each tube, and the contents were mixed via vortexing for one minute. Each tube was then centrifuged (Microfuge 2, Beckman Coulter, CA, USA) at 18,000× *g* for 2 min at 4 °C. After centrifugation, the supernatant was carefully decanted, and the tubes centrifuged again at 18,000× *g* for 2 min at 4 °C. Following this second centrifugation procedure, the residual supernatant was carefully removed by pipetting, ensuring the bacterial pellet was not disturbed. The tubes containing the bacterial pellet were then left uncovered and allowed to air-dry at room temperature.

Following air-drying, 10 µL of 70% of Formic Acid (Fluka, Sigma Aldrich, St. Louis, MO, USA) was added to each tube, and the contents were agitated via vortexing for 1 min to ensure the resuspension of the bacterial pellets. The suspensions were then left to stand for 5 min, after which, 10 µL of HPLC-grade acetonitrile (Fischer Scientific, Fair lawn, NJ) was added to each tube and mixed thoroughly via vortexing. The tubes were then centrifuged at 18,000× *g* for 2 min at 4 °C.

After centrifugation, 1 µL of the supernatant was pipetted without disturbing the pellet and transferred to one well on the MSP 96 polished-steel target (Bruker Daltonics, Billerica, MA, USA). This was done in triplicate for each sample. The 1 µL aliquots of supernatant on the target were allowed to air-dry at room temperature, following which each well was overlayed with 1 µL of Bruker Matrix HCCA (10 mg/mL) solution (α-Cyano-4-hydroxycinnamic acid, Bruker Daltonics, Billerica, MA, USA), which had been reconstituted in 250 µL of organic solvent (comprising acetonitrile, trifluoracetic acid, and water). The steel target was then allowed to air-dry. The mass spectrum of each protein extract was then assessed using Bruker Microflex LT (Bruker Daltonics, Billerica, MA, USA), and a reference Mass Spectral Profile (MSP) was created as described below.

**Mass Spectral Profile Creation:** Prior to analyzing the mass spectra of protein extracts, each target was calibrated to ensure the accuracy of the measurements. Calibration was achieved by placing 1 µL of Bacterial Test Standard (BTS; Bruker Daltonics, Billerica, MA, USA) in one empty well of the target containing the protein extract samples. Once dried, this well was then overlayed with HCCA matrix as described above, and the auto-calibration option in the flexControl software (Bruker Daltonics, Billerica, MA, USA) was used to facilitate calibration. Once calibrated, spectra were acquired using the AutoXecute function within flexControl. Three spectra were recorded from each sample well, and there were 15 replicates of each serovar; thus, each reference spectrum was constructed using 45 spectra. The spectra were obtained using the recommended setting for bacterial species identification (linear positive mode, 20 Hz laser frequency, 20 kV acceleration voltage, 18.5 kV IS2 voltage, 250 ns extraction delay).

Following acquisition, all spectra for each serotype were then imported into flexAnalysis (Bruker Daltonics, Billerica, MA, USA). The spectra were subjected to baseline subtraction and mass spectrum smoothing for evaluation of their uniformity and detection of abnormal peaks, flatlines, or other anomalies. The identity of any discrepant spectra was recorded, and these spectra were removed from further analysis. All spectra passing the previous quality control screening were then imported into MALDI Biotyper 3.0 (Bruker Daltonics, Billerica, MA, USA), and the new MSP for each serotype was created. Additionally, the peak list was evaluated to ensure there were a minimum of 70 peaks per MSP and these peaks were in 90% or greater of the constituting spectra. All six MSPs were then added to create a new reference library.

**Isolate identification using MALDI-TOF:** The unknown *Salmonella* isolates recovered from the survival studies at each location were shipped to the OARDC, where they were cultured and processed for Intact Cell Mass Spectrometry (ICMS). Briefly, one loopful of the previously frozen culture broth was streaked for colony isolation onto LB agar plates supplemented with rifampicin (80 µg/mL) and incubated at 37 °C overnight. The following day, one individual colony was selected and smeared onto two separate wells of the MALDI-TOF target. The wells were then overlayed with 1 µL of HCCA matrix, allowed to air-dry at room temperature, and analyzed using Bruker Microflex LT (Bruker Daltonics, Billerica, MA, USA), with the instrument settings described earlier. Identification of the samples was done automatically using Bruker Realtime Classification software (Bruker Daltonics, Billerica, MA, USA) by comparing the isolate MSP with the 6 newly created reference MSP.

**Isolate identification using Serology and PCR:** The identities of the isolates were confirmed via a combination of serotyping of somatic antigens and a PCR-based assay. This was done independently of the MALDI-TOF analysis to ensure there was no perceived bias. The unknown isolates were streaked for colony isolation onto LB agar plates supplemented with rifampicin (80 µg/mL) and incubated at 37 °C overnight. The next day, one individual colony was selected from each plate and mixed with 10 µL of *Salmonella* antisera^k^ for somatic groups B, C1, C2, D, and E1 and observed for agglutination. Two of the serotypes, *S.* Montevideo and *S.* Braenderup, belong to Group C1 and agglutinated equally when mixed with C1 antisera. The differentiation of these two serotypes was accomplished via a PCR-based assay adapted from J. Jean-Gilles Beaubrun et al. (2012) [17]. Briefly, individual colonies from samples agglutinating with C1 were placed in 1.7 mL microcentrifuge tubes containing 100 µL of sterile DNAase-free water. The tubes were then placed in a water bath at 100 °C for 10 min. The cell lysates were then used as DNA templates for PCR screening for the detection of *stm1350* (171 bp) and *sty0346* and *sty0347* (262 bp) [17]. The primer sequences used were *stm1350F*: 5′TCAAAATTACCGGGCGCA3′; *stm1350R:* 5′TTTTAAGACTACATACGCGCATGAA3′; *STY0346:* 5′GGCTGGAGCAGCCTTACAAAA3′; and *sty0347* 5′AAGAGTTGCCTGGCTGGTAAAA3′. Amplifications were performed in a 50 μL reaction mixture containing 25 μL GoTaq Green Mastermix (Fischer Scientific, Fair lawn, NJ, USA), 2 μL *sty0346* (5 μM/μL), 2 µL *sty0347* (5 μM/μL), 2 μL *stm1350* F (5 μM/μL), 2 μL *stm1350* R (5 μM/μL), 14 μL H_2_O, and 3 μL DNA template. The reaction mixture was heated to initial denaturation at 94 °C for 5 min, followed by 35 cycles at 94 °C for 30 s, 52 °C for 1 min, 72 °C for 1 min, and final extension at 72 °C for 5 min. The PCR products were separated on 2% agarose gels stained with ethidium bromide and visualized using UV light.

**Data Analysis:** The results were tabulated using 2 × 2 contingency tables for each serotype to reflect the agreement between the results obtained from MALDI-TOF and those from serotyping/PCR. The overall percent agreement (overall number of isolates positively and negatively identified as a particular by both MALDI TOF and Serology/Number of isolates tested), positive percent agreement (Number Samples positively identified as a particular serotype by both MALDI-TOF and Conventional Serology/Number of Samples identified as the serotype using conventional serology), negative percent agreement (Number Samples identified as not being a particular serotype by both MALDI-TOF and Conventional Serology/Number of Samples identified as the serotype using conventional serology), and Cohen’s kappa were calculated to evaluate the agreement between the methods. Cohen’s Kappa provided a measure of the degree to which the two methods concurred in their respective classification of the different serovars [18,19]. Statistical calculations were done using VassarStats [20].

## 3. Results

**Spectra Validation**: To validate the accuracy of the created MSPs, 40 samples of each serotype whose identity was known were run against the newly created MSP library. All correctly identified reference isolates had an overall mean score for all serotypes of 2.54 (95% CI: 2.52–2.56). For the identification and subtyping of the isolates, we conservatively used the lower limit of 2.52 as our cut-off. If an unknown isolate achieved a biotyper score of less than 2.52 for the best match when analyzed, the isolates were restreaked, and fresh colonies were reanalyzed.

*Salmonella* subtyping: MALDI-TOF subtyped 95% (803/845) of *Salmonella* isolates the same as the conventional serotyping and PCR combination. The majority of isolates recovered, 430, belonged to the serovar *S.* Javiana, while the least number of recovered isolates, 17, belonged to the *S.* Newport serovar (Table 1). The positive percent agreement, which is a measure of MALDI-TOF ability to identify a serovar compared to the conventional methods, was lowest for *S.* Montevideo (79%) and highest for *S.* Typhimurium and *S.* Newport, where 100% of isolates were correctly matched. The negative percent agreement, which is a measure of MALDI-TOF ability to correctly classify a sample as not being a particular serotype, was above 98% for all serotypes.

We also calculated Cohen’s Kappa as another measure to evaluate the agreement between the subtyping results obtained by MALDI-TOF and by the conventional serotyping/PCR method. The subtyping of *S.* Newport received the lowest Cohen’s Kappa score (0.85), while the subtyping of *S*. Javiana received the highest score (0.98) (Table 1).

## 4. Discussion

Herein, we report that MALDI-TOF is capable of subtyping *Salmonella* using mass spectral profile analysis equally as well as conventional methods (serotyping + PCR), while at the same time allowing a rapid identification at a reduced cost. We can thus propose that MALDI-TOF can be used as an alternative method for the rapid identification of *Salmonella* serovars used in epidemiological studies such as this one.

Surprisingly, although MALDI-TOF technology has been in existence for over 20 years, there is a paucity of published literature describing its ability and use to identify *Salmonella* serovars. At the species level, *Salmonella enterica* subspecies enterica was first differentiated from other *Salmonella enterica* subspecies using mass spectrometry in 2008 [21]. Serovar differentiation capability was also first reported by Dieckmann et al. in 2011, when he reported successful ICMS differentiation of *S.* Enteriditis, *S.* Typhimurium, *S.* Virchow, *S.* Infantis, and *S*. Hadar [9]. The authors further postulated the existence of potential discriminatory biomarker ions in *Salmonella enterica* serotypes, but these have not been extensively evaluated. Discrimination at this phylogenic level was later demonstrated by Kuhns et al., (2012), who were able to differentiate *Salmonella* Typhi from other serovars, but not to differentiate between the other 11 serovars of *Salmonella enterica* subspecies *enterica* tested [22]. Discrimination between bacterial serovar strains may be required in disease outbreaks, especially foodborne disease outbreaks. While this strain discrimination is possible for certain bacterial species, this has not been the case for *Salmonella* species using conventional MALDI-TOF [23]. Additional research is required to evaluate to potential for MALDI-TOF MS combined with principal component analysis or MALDI-TOF MS–MS to provide this level of discriminatory power, especially if MALDI-TOF is to be used in foodborne disease outbreak investigations [13,24]. With conventional MALDI-TOF analysis, the reproducibility of unique strain specific biomarker peaks necessary for *Salmonella* spp. strain identification has historically posed a problem, but recent studies have postulated the reproducibility of these peaks may be increased by controlling bacterial concentration, using the supernatant obtained after centrifuging the colony suspension, and including various matrix additives [25]. The potential to identify both serovar- and strain-specific biomarker peaks will undoubtedly increase the usefulness and precision of MALDI-TOF in clinical and epidemiological investigations.

MALDI-TOF biotyper scores are assigned based on the similarity of an organism’s mass spectrum to the reference spectra [14]. The quality of the spectra can be dependent on a number of factors including age of culture, sample preparation, thickness of colony smear on target, and matrix used [26,27,28]. The difference between the methods used for the creation of the reference MSP and the analysis of unknown isolates, i.e., protein extraction vs. intact cell mass spectrometry (ICMS), can potentially affect the identification of organisms [29]. This effect is more pronounced with Gram-positive organisms and yeast cells, whose the thicker cell wall may affect identification when the direct smear method is used [30,31]. For Gram-negative bacteria, such as *Salmonella* spp., this is less pronounced and may only lead to discordant results in a small number of isolates; consequently, the direct smear approach for the analysis of these isolates, which is faster and more economical, is suitable [30,32]. However, in cases where strain identification is necessary, for example in foodborne disease outbreaks, it may be necessary to use the protein extraction technique to enhance the expression of biomarker peaks in the analyte and thus improve the accuracy of the identification. Ford et al. (2013) also reported that rate of identification dropped as the culture age increased, and Veelo et al., (2014) further reported that thickness of the smear on the target can affect the identification of organisms [27,33]. It is for these reasons, if isolates obtained low scores below our cut-off values, that we repeated the culture and smear preparation one more time and reanalyzed the isolates with reference strains to validate the preparation procedures.

Biotyper scores are logarithmic, ranging between 0 and 3, and high values in this score represent a high similarity with the database entry [34]. Currently, MALDI-TOF scores greater than 2.3 are suggested by the manufacturers to indicate a high probability of species identification. However, for this experiment, a higher discriminatory power for serovar identification is required, and no MALDI-TOF scores are suggested for this level of discrimination. Consequently, since we required an increased similarity match for serovars versus that normally required for species identification, we increased our serovar classification score above the value of 2.3 suggested for species identification and used a score based on screening of the reference spectra against the newly created *Salmonella* library as our cut-off. Adjustment to biotyper cut-off scores is not unique, and other authors have also proposed adjustments to biotyper classification cut-off scores to optimize the classification of organisms, but these studies were confined to improve genus and species classification [35,36,37] and not serovar classification, as we showed.

Although the initial cost of MALDI-TOF machines can be high, the money saved from the cost of diagnostic reagents and decreased labor requirements, coupled with the rapid identification time, makes MALDI-TOF identification of bacteria a suitable alternative to conventional organism identification methods [14]. These benefits are most pronounced in epidemiological studies like this one, where large numbers of samples are processed. For example, the average cost of consumables used for serotyping a *Salmonella* isolate is estimated to be USD 8.00 [38], while consumables used in MALDI-TOF costs an estimated USD 0.50 [14,39]. The difference in processing time was also very apparent, since the identification of a *Salmonella* isolates using the combination of serotyping and PCR took at most 3 h for some isolates, while MALDI-TOF identification took as little as 3 min per isolate, including the time necessary for sample preparation and MALDI-TOF analysis.

In conclusion, the MALDI-TOF method proposed here as a rapid, cost-effective method for the identification of *Salmonella* serovars was proven to have equal diagnostic capabilities as conventional subtyping methods. Consequently, this method can be used to complement conventional methods of serovar identification; however, if definitive serovar identification and strain discrimination are required, as in the case of outbreak scenarios, the identification of isolates will still rely on molecular confirmatory tests.

## Figures and Tables

**Table 1:** Comparison of the results obtain from MALDI-TOF and conventional subtyping methods.

Salmonella Serotype *	Number ** of Isolates	MALDI–TOF ***						OPA §	PPA †	NPA ‡	Cohen’s Kappa #
		S.T	S.M	S.A	S.J	S.B	S.N				
S.T	38	38						98.6%	100%	98.5%	0.86
S.M	48	5	38	4	1			98.7%	79.1%	99.8%	0.87
S.A	156	5		144		2	5	96.8%	92.3%	97.8%	0.89
S.J	430		1		429			99.0%	98.8%	99.0%	0.98
S.B	154			11	3	137	1	97.7%	89.0%	99.7%	0.92
S.N	17						17	99.3%	100%	99.3%	0.85

* S.T—*Salmonella* Typhimurium, S.M—*Salmonella* Montevideo, S.A—*Salmonella* Anatum, S.J—*Salmonella* Javiana, S.B.—*Salmonella* Braenderup, S.N—*Salmonella* Newport; ** Number of isolates identified using conventional serology/PCR; *** Number of isolates identified using MALDI-TOF; I§ OPA—Overall Percent Agreement; † PPA—Positive Percent Agreement; ‡ NPA—Negative Percent Agreement; # Kappa scores greater than 0.81 are considered to indicate an almost perfect agreement between the testing methods [18,19].

## Data Availability

The data that support the findings of this study are available on request from the corresponding author. The data are not publicly available due to privacy restrictions.

## References

[1] Lee K.-M., Runyon M., Herrman T.J., Phillips R., Hsieh J. (2015). Review of Salmonella detection and identification methods: Aspects of rapid emergency response and food safety. Food Control.

[2] Scallan E., Hoekstra R.M., Angulo F.J., Tauxe R.V., Widdowson M.A., Roy S.L., Jones J.L., Griffin P.M. (2011). Foodborne illness acquired in the United States-major pathogens. Emerg. Infect. Dis..

[3] Baird-Parker A.C. (1990). Originally published as Volume 336, Issue 8725Foodborne salmonellosis. Lancet.

[4] Sabat A., Budimir A., Nashev D., Sá-Leão R., Van Dijl J., Laurent F., Grundmann H., Friedrich A., Markers E.S.G.o.E. (2013). Overview of molecular typing methods for outbreak detection and epidemiological surveillance. Euro Surveill. Bull. Eur. Sur Les Mal. Transm.= Eur. Commun. Dis. Bull..

[5] Carbonnelle E., Mesquita C., Bille E., Day N., Dauphin B., Beretti J.L., Ferroni A., Gutmann L., Nassif X. (2011). MALDI-TOF mass spectrometry tools for bacterial identification in clinical microbiology laboratory. Clin. Biochem..

[6] Wattiau P., Boland C., Bertrand S. (2011). Methodologies for Salmonella enterica subsp. enterica subtyping: Gold standards and alternatives. Appl. Environ. Microbiol..

[7] https://www.academia.edu/download/41403599/Antigenic_Formulae_of_the_Salmonella_ser20160122-19332-1wjmz5c.pdf.

[8] McQuiston J.R., Fields P.I., Tauxe R.V., Logsdon J.M. (2008). Do Salmonella carry spare tyres?. Trends Microbiol..

[9] Dieckmann R., Malorny B. (2011). Rapid screening of epidemiologically important Salmonella enterica subsp. enterica serovars by whole-cell matrix-assisted laser desorption ionization-time of flight mass spectrometry. Appl. Environ. Microbiol..

[10] Schrader K.N., Fernandez-Castro A., Cheung W.K., Crandall C.M., Abbott S.L. (2008). Evaluation of commercial antisera for Salmonella serotyping. J. Clin. Microbiol..

[11] Kliem M., Sauer S. (2012). The essence on mass spectrometry based microbial diagnostics. Curr. Opin. Microbiol..

[12] Christner M., Trusch M., Rohde H., Kwiatkowski M., Schluter H., Wolters M., Aepfelbacher M., Hentschke M. (2014). Rapid MALDI-TOF mass spectrometry strain typing during a large outbreak of Shiga-Toxigenic Escherichia coli. PLoS ONE.

[13] Sandrin T.R., Goldstein J.E., Schumaker S. (2013). MALDI TOF MS profiling of bacteria at the strain level: A review. Mass Spectrom. Rev..

[14] Dingle T.C., Butler-Wu S.M. (2013). Maldi-tof mass spectrometry for microorganism identification. Clin. Lab. Med..

[15] Topalcengiz Z., Spanninger P.M., Jeamsripong S., Persad A.K., Buchanan R.L., Saha J., LeJEUNE J., Jay-Russell M.T., Kniel K.E., Danyluk M.D. (2020). Survival of Salmonella in various wild animal feces that may contaminate produce. J. Food Prot..

[16] Schumann P., Maier T. (2014). MALDI-TOF mass spectrometry applied to classification and identification of bacteria. Methods Microbiol..

[17] Jean-Gilles Beaubrun J., Cheng C.M., Chen K.S., Ewing L., Wang H., Agpaoa M.C., Huang M.C., Dickey E., Du J.M., Williams-Hill D.M. (2012). The evaluation of a PCR-based method for identification of Salmonella enterica serotypes from environmental samples and various food matrices. Food Microbiol..

[18] Landis J.R., Koch G.G. (1977). An application of hierarchical kappa-type statistics in the assessment of majority agreement among multiple observers. Biometrics.

[19] Landis J.R., Koch G.G. (1977). The measurement of observer agreement for categorical data. biometrics.

[20] http://vassarstats.net/.

[21] Dieckmann R., Helmuth R., Erhard M., Malorny B. (2008). Rapid classification and identification of salmonellae at the species and subspecies levels by whole-cell matrix-assisted laser desorption ionization-time of flight mass spectrometry. Appl. Environ. Microbiol..

[22] Kuhns M., Zautner A.E., Rabsch W., Zimmermann O., Weig M., Bader O., Gross U. (2012). Rapid discrimination of Salmonella enterica serovar Typhi from other serovars by MALDI-TOF mass spectrometry. PLoS ONE.

[23] Kang L., Li N., Li P., Zhou Y., Gao S., Gao H., Xin W., Wang J. (2017). MALDI-TOF mass spectrometry provides high accuracy in identification of Salmonella at species level but is limited to type or subtype Salmonella serovars. Eur. J. Mass Spectrom..

[24] Yan W., Qian J., Ge Y., Ye K., Zhou C., Zhang H. (2020). Principal component analysis of MALDI-TOF MS of whole-cell foodborne pathogenic bacteria. Anal. Biochem..

[25] Fukuyama Y., Ojima-Kato T., Nagai S., Shima K., Funatsu S., Yamada Y., Tamura H., Nomura S., Ogata K., Sekiya S. (2019). Improved MALDI-MS method for the highly sensitive and reproducible detection of biomarker peaks for the proteotyping of Salmonella serotypes. J. Mass Spectrom..

[26] Giebel R., Worden C., Rust S.M., Kleinheinz G.T., Robbins M., Sandrin T.R. (2010). Microbial fingerprinting using matrix-assisted laser desorption ionization time-of-flight mass spectrometry (MALDI-TOF MS) applications and challenges. Adv. Appl. Microbiol..

[27] Ford B.A., Burnham C.A. (2013). Optimization of routine identification of clinically relevant Gram-negative bacteria by use of matrix-assisted laser desorption ionization-time of flight mass spectrometry and the Bruker Biotyper. J. Clin. Microbiol..

[28] Holland R.D., Wilkes J.G., Cooper W.M., Alusta P., Williams A., Pearce B., Beaudoin M., Buzatu D. (2014). Thymol treatment of bacteria prior to matrix-assisted laser desorption/ionization time-of-flight mass spectrometric analysis aids in identifying certain bacteria at the subspecies level. Rapid Commun. Mass Spectrom RCM.

[29] Cuénod A., Foucault F., Pflüger V., Egli A. (2021). Factors associated with MALDI-TOF mass spectral quality of species identification in clinical routine diagnostics. Front. Cell. Infect. Microbiol..

[30] Bizzini A., Durussel C., Bille J., Greub G., Prod’Hom G. (2010). Performance of matrix-assisted laser desorption ionization-time of flight mass spectrometry for identification of bacterial strains routinely isolated in a clinical microbiology laboratory. J. Clin. Microbiol..

[31] Fournier R., Wallet F., Grandbastien B., Dubreuil L., Courcol R., Neut C., Dessein R. (2012). Chemical extraction versus direct smear for MALDI-TOF mass spectrometry identification of anaerobic bacteria. Anaerobe.

[32] Mestas J., Quias T., Dien Bard J. (2016). Direct Identification of Aerobic Bacteria by Matrix-Assisted Laser Desorption Ionization Time-of-Flight Mass Spectrometry Is Accurate and Robust. J. Clin. Lab. Anal..

[33] Veloo A.C., Elgersma P.E., Friedrich A.W., Nagy E., van Winkelhoff A.J. (2014). The influence of incubation time, sample preparation and exposure to oxygen on the quality of the MALDI-TOF MS spectrum of anaerobic bacteria. Clin. Microbiol. Infect. Off. Publ. Eur. Soc. Clin. Microbiol. Infect. Dis..

[34] Richter C., Hollstein S., Woloszyn J., Kaase M., Gatermann S.G., Szabados F. (2012). Evaluation of species-specific score cut-off values for various Staphylococcus species using a MALDI Biotyper-based identification. J. Med. Microbiol..

[35] Schmitt B.H., Cunningham S.A., Dailey A.L., Gustafson D.R., Patel R. (2013). Identification of anaerobic bacteria by Bruker Biotyper matrix assisted laser desorption ionization-time of flight mass spectrometry with on-plate formic acid preparation. J. Clin. Microbiol..

[36] Schulthess B., Bloemberg G.V., Zbinden R., Böttger E.C., Hombach M. (2014). Evaluation of the Bruker MALDI Biotyper for Identification of Gram-Positive Rods: Development of a Diagnostic Algorithm for the Clinical Laboratory. J. Clin. Microbiol..

[37] Barberis C., Almuzara M., Join-Lambert O., Ramírez M.S., Famiglietti A., Vay C. (2014). Comparison of the Bruker MALDI-TOF Mass Spectrometry System and Conventional Phenotypic Methods for Identification of Gram-Positive Rods. PLoS ONE.

[38] Association of Public Health Laboratories (2014). Salmonella Serotyping in US Public Health Laboratories. http://www.aphl.org/AboutAPHL/publications/Documents/FS_SalmonellaSustainabilityWhitePaper_Nov2014.pdf.

[39] Singhal N., Kumar M., Kanaujia P.K., Virdi J.S. (2015). MALDI-TOF mass spectrometry: An emerging technology for microbial identification and diagnosis. Front. Microbiol..

